# Mobile dental clinic for oral health services to underserved rural Indian communities

**DOI:** 10.6026/973206300191383

**Published:** 2023-12-31

**Authors:** Renu Bala, Vinod Sargaiyan, Sneha Amit Rathi, Sonika Samrat Mankar, Anshul Kishor Jaiswal, Samrat Ashok Mankar

**Affiliations:** 1 Department of Periodontology, PDM Dental College and Research Institute, Bahadurgarh, Haryana, India; 2 Department of Oral Pathology & Microbiology, Maharana Pratap College of Dentistry & Research Centre, Gwalior, M.P., India; 3 Department of Prosthodontics and Crown & Bridge, Dr HSRSM dental college, Hingoli, Maharashtra, India; 4 Department of Prosthodontics and Crown & Bridge, Dr HSRSM dental College, Hingoli, Maharashtra, India; 5 Department of Prosthodontics and Crown & Bridge, Dr HSRSM dental College and hospital, Hingoli, Maharashtra, India; 6 Department of Prosthodontics and Crown & Bridge, Dr. HSRSM dental college, Hingoli, Maharashtra, India

**Keywords:** Mobile dental program, oral care, underserved rural communities

## Abstract

Mobile dental vehicle (MDVs) can be adopted to address the oral healthcare needs of different populations. Therefore, it is of interest to evaluate mobile dental clinic program in providing oral health services to underserved rural communities. A 2 x 2.5 meter box trailer is used by the UN, and it can be carried by a vehicle with all-wheel drive for all types of weather. There was a small box trailer with its weather proof canvas cover extended, changing it into four dental operators and an integrated waiting and teaching space. Clinical examination, scaling, polishing, health education, individual and group teaching in dental hygiene, fluoride applications, fissure sealants, amalgam and composite restorations, extractions, and minor oral surgery were all supplied at no cost to the patients. In a longitudinal study of 3 years in underserved rural areas, a total of 6326 patients were provided different dental treatments. It was found that 93.3% patients did not undergo any dental treatment in the past.

## Background:

The urban-rural gap in oral health is large, and oral illnesses continue to be a top public health concern globally [1, 2]. However, there are several obstacles that make it hard for people living in rural regions to get the oral health treatment they need, despite the fact that they have a greater frequency and severity of dental caries and periodontal disease [3,4]. Traditional approaches, such as constructing dental clinics and hospitals, or providing outreach treatments using disposable materials, are impractical and inefficient in remote areas. In many areas, conventional oral health care is lacking [5,6]. Efforts to narrow the gap in access to dental care between urban and rural areas have been advocated. All of these approaches may help overcome these challenges to varying degrees, but none of them are fully applicable in rural settings [7, 8]. If dental clinics could be established in remote regions with the necessary facilities, that would be great. However, it may be difficult to attract dental professionals to these remote clinics due to the high expense of starting a dental practice and the possibility of unreliable or non-existent electricity and water supplies [9, 10]. In addition, the low population density means that the clinic is underutilized most of the time, resulting in extremely poor cost-effectiveness [11,12].

People who are homeless, displaced, migrants, residents of rural or distant locations, residents of poor socioeconomic neighborhoods, and others who cannot afford regular dental treatment may benefit from Mobile Dental Vehicle (MDVs) that bring dental services to them [13,14]. A commercial motor vehicle may be anything from a truck to a bus. The original model of this MDV was a conventional bus that was upgraded [15, 16]. There's one section for the generators, another for the drivers, a reception desk and waiting room, and a separate room for dentistry work. In order to meet the electrical demands of the MDV's lighting and dental clinic, It features a generator set that can produce three-phase electricity. The MDV is supplied with liquids from three separate tanks: fresh water, waste water, and recycling water [17,18]. There is potable water for medical usage in the tank. Waste water is collected in the drain water tank until it can be emptied into a proper drainage system. Suction for the clinical aspirator is generated by a moving current generated by the perpetually circulating water in the recycling water tank [19,20].

Dentists are not required to permanently relocate to rural areas in order to participate in the MDV program. Part-time MDV service is available to urban-based dentists, especially those who are completing post-doctoral residency programs in community dentistry [21,22]. As a result of this agreement, the underserved population will have access to professional assistance and guidance on a consistent basis; this will help alleviate the problem of a lack of workers in rural regions [23,24]. There has been no study that has been carried out to evaluate the mobile dental program in providing oral health services to underserved rural areas. Therefore, it is of interest to discuss the use of MDVs as a solution to urban-rural inequality in receiving oral healthcare. The results of this study will help policy makers in adopting MDV for rural areas in broader scale.

## Materials and Method:

## Evolution and description of the mobile dental unit:

India has a big population that is underserved in terms of dental care, and the region's topography may be rough and difficult to navigate. Unique in its mechanical design, the unit was developed in tandem with the Council for Scientific and Industrial Research. In addition, we sought the expertise of dental equipment distributors and their technical support teams. A 2 x 2.5 meter box trailer is used by the UN, and it can be carried by a vehicle with all-wheel drive in all weather. There was a small box trailer with its weatherproof canvas cover extended, changing it into four dental operatories and an integrated waiting and teaching space. A group of electrically powered arms are triggered by a remote to carry out the deployment. Once the cover is set up, guy ropes are used to keep it in place. Dental consoles are conveniently located on the unit's ceiling-mounted delivery rails on both sides. When the consoles are pulled out of their storage, they allow for simultaneous treatment of four patients. Each workstation may be run in either a four-person or single-person mode. The third and last section of the building is located at the back, and it serves as a waiting room and health education center. It has a TV screen, a video recorder, a slide projector, and a library of slide shows. A variety of health education posters and leaflets are also available in the unit's many languages. In the waiting room/health education space, there are four patient sofas, eight operator/assistant seats, and ten foldable chairs, as well as a central suction system and a generator. Instruments, supplies, medications, and patient record cards may all be stored in cupboards and on work tables. The facility has four fully-equipped dental surgeries' worth of equipment and supporting materials. Once deployed, it stays there until all patients have been cared for, and the whole process takes no more than 45 minutes from arrival to first treatment. An alarm system has been installed for heightened safety. We had one project coordinator, two dental therapists, one chairside assistant, and two oral hygienists on staff while the facility was in operation.

## Evaluation of different dental clinical procedures carried out in underserved rural areas using MDV:

It was anticipated, based on prior experience in dentally deprived communities, that the demand for treatment would considerably outstrip the capacity of the personnel. So, in both suburban and urban settings, certain populations were singled out for special attention. When other patients needed help, it was given to them immediately. Rural regions specifically targeted nurses, teachers, and students, with the unit located at various health care facilities. In metropolitan locations, students and educators from particular schools were chosen as participants because to the overwhelming need for treatment and prevention programs that had been shown via earlier engagement. Exams, scaling, polishing, health education, individual and group teaching in dental hygiene, fluoride applications, fissure sealants, amalgam and composite restorations, extractions, and minor oral surgery were all supplied at no cost to the patients. The facility not only filled a critical role, but also served as a testing ground for several studies and a teaching tool for students. This initiative helps students put into practice the ideals of community service and get valuable clinical experience. Students get an understanding of the unique health concerns of persons from diverse backgrounds.

## Statistical analysis:

The findings were represented in the form of percentages. The SPSS version 21 was used for statistical analysis. The ANOVA and chi square test was used for statistical analysis.

## Results:

In a 3 longitudinal study carried out in underserved rural areas, a total of 6326 patients were provided different dental treatments. It was found that 93.3% patients did not undergo any dental treatment in the past. 52.14% patients were males while 47.86 % patients were females. 11.14% patients were up to 16 years of age. 13.27% patients were in the age group of 17-25 years. 22.54% were in 26-45 years, 30.12% in 46-67 years and 22.93% in 68-85 years of age. Most of the patients were from the older age group (Table 1). 2532 patients (39%) underwent oral examinations including clinical diagnosis of premalignant lesions and conditions. Scaling and polishing was carried out in 602 (10%) patients. Fissure sealants were applied in 30 (0.5%) patients. Amalgam restorations were applied in 354 (6%) patients. Composite restoration was carried out in 64 (1%) patients. Temporary restorations were carried out in 68 (1%) patients. Extractions were carried out in 2578 (42%) patients. Fluoride treatment was provided in 34 (0.5%) patients. The maximum dental treatment carried out was extractions followed by oral examination including premalignant and malignant lesions and conditions. (p= 0.001) Least dental procedures carried out were composites and temporary fillings (Table 2).

## Discussion:

In remote locations, doctors, nurses, and other medical professionals might be hard to serve [24, 25]. Due to a small patient pool spread over a vast region, rural clinics have a hard time attracting and retaining dentists and paradental staff, and because rural places are not often seen as offering as many work chances or as promising of a future [26,27]. In many areas, conventional oral health care is lacking, hence mobile dental vans (MDVs) have been advocated as a complementary option [14,15]. Typically, they have been used in programs to check the general community for oral disorders, provide dental treatment to the homeless and migratory populations, and promote oral health in schools. MDVs are especially helpful for rural locations with underserved populations because of their portability [16,17]. The development of dental tools has made it possible for MDVs to function independently. This means that the MDV can perform practically all of the same functions as a regular dentist office, such as dental scaling, filling, and extraction [18,19]. This article discusses how mobile dental vans (MDVs) might help bridge the gap in dental care availability between urban and rural areas. A study [23] evaluated the cost-effectiveness of a proposal for a dental health initiative centered on schools. The excess was utilized to raise payments to community clinics and private practices for treating children, and mobile/portable technology was used by hygienists to screen and prevent illness among pupils, a move that has the potential to significantly equalize access for all children. Another study [26] was conducted to evaluate mobile dental clinic van for routine cleanings and more extensive procedures. Eight cleanings, seven fillings, two sealants, and one extraction or referral were performed daily, on average. Programs that bring dentists to patients in mobile clinics have raised the profile of dental schools on campus and in the surrounding community. A trial was carried out [27] in Southern Africa region found that there is a significant need for dental care in areas that have been historically neglected.

Some researchers [28] studied a large number of kids, focusing on those from disadvantaged backgrounds. Many communities have turned to mobile dentistry clinics as a solution to their problems. Three mobile programs in Connecticut were issued structured questionnaires to gather data on their ages, challenges in planning and execution, continuing expenses, and productivity. A study [29] was performed to evaluate the oral health of migrant children, how to provide them dental care, and how mobile clinics may help. It was found that migrant children had significant dental care requirements, and that these clinics on wheels were there to help. Although helpful in providing therapy, the service's effectiveness was hindered by a few issues. Because of the long commute times and the short school day, clinical work in schools was typically cut down to just three hours a day in metropolitan regions [23,124]. At first, there were a number of missed workdays owing to technical and mechanical issues. This is in line with the findings from similar programs using mobile dental units [25,26]. However, the unit's track record has proved that it is a feasible approach to deliver dental treatment, and the overall results have been good. As a result, it has been able to deliver high-quality primary oral care to underserved populations via the effective employment of teams of oral health auxiliary workers including those in rural areas, students from disadvantaged schools, and those with mental and physical disabilities [27,28].

A higher share of the elderly lives in rural regions since the younger generation often migrates to urban centers in search of better educational and employment prospects [11,12]. The physical health of this population makes it more difficult for them to access oral healthcare; they may not have as much access to information about oral care as their urban colleagues, who may be more systematically cared for in elderly homes and institutions [13,14]. In most cases, in rural areas, population density is minimal. Due to the low population density and physical isolation typical of rural areas, most residents cannot afford routine oral health treatments [15,16]. Residents in these locations may be eager to drive to the closest dental clinic, but they may not have access to reliable public transit, and they may face hazardous driving conditions due to poor infrastructure. Due to these factors, it may be challenging for rural inhabitants, especially those with little financial resources, to make the trip to the closest clinic or medical facility in order to get oral healthcare [17,18]. Dental care may be brought to areas with poor access to public transportation thanks to the portability of an MDV. It is especially useful for service delivery in sparsely populated regions to occasionally organize for visits to many locations through multiple routes [19, 20]. To ensure that high-tech dental care can be provided in areas with unreliable power and water, An MDV's standard equipment includes a generator set for 24/7 power, water tanks, and plumbing [21, 22]. Because of its accessibility features, MDVs can transport people who use wheelchairs. Dental treatment may be provided by an MDV stationed close to a person's place of residence for those who are unable to make the long trip to a traditional office [23,24]. MDVs have been advocated as a complementary option. Typically, they have been used in programs to check the general community for oral disorders, provide dental treatment to the homeless and migratory populations, and promote oral health in schools [25, 26]. MDVs are especially helpful for rural locations with underserved populations because of their portability. The development of dental tools has made it possible for MDVs to function independently [27, 28]. This means that the MDV can perform practically all of the same functions as a regular dentist office, such as dental scaling, filling, and extraction.

## Conclusion:

In 3 years, a total of 6326 patients were provided different dental treatments by MDV. It was found that 93.3% patients did not undergo any dental treatment in the past. Although helpful in providing therapy, the MDV service's effectiveness was hindered by a number of logical issues.

## Figures and Tables

**Table 1:** Demographic details of total patients treated by MDV

**Total patients evaluated**	**6326**
**Past history of dental visit**	
Yes	6.70%
No	93.30%
Gender	
Male	52.14%
Female	47.86%
Age groups	
Upto 16 years	11.14%
17-25 years	13.27%
26-45 years	22.54%
46-67 years	30.12%
68-85 years	22.93%

**Table 2:** Details of different dental clinical procedures carried out in underserved rural areas using MDV.

	**No**	**%**	**P-value**
Oral examination (premalignant and malignant lesions)	2532	39	0.001*
Scaling and polishing	602	10	
Fissure sealants	30	0.5	
Amalgams	354	6	
Composites	64	1	
Temporary fillings	68	1	
Extractions	2578	42	
Fluoride treatments	34	0.5	
Total	6326	100	
